# Invasive Aspergillosis Mimicking Metastatic Lung Cancer

**DOI:** 10.3389/fonc.2018.00188

**Published:** 2018-06-05

**Authors:** Michiel J. E. G. W. Vanfleteren, Anne-Marie C. Dingemans, Veerle F. Surmont, Karim Y. Vermaelen, Alida A. Postma, Astrid M. L. Oude Lashof, Cordula C. M. Pitz, Lizza E. L. Hendriks

**Affiliations:** ^1^Department of Respiratory Medicine, University Hospital Ghent, Ghent, Belgium; ^2^Department of Respiratory Medicine, Maastricht University Medical Centre (MUMC+), Maastricht, Netherlands; ^3^Department of Respiratory Medicine, Sint-Jozefskliniek Izegem, Izegem, Belgium; ^4^Department of Radiology and Nuclear Medicine, Maastricht University Medical Centre (MUMC +), Maastricht, Netherlands; ^5^Department of Medical Microbiology, Section Infectious Diseases, Maastricht University Medical Centre (MUMC +), Maastricht, Netherlands; ^6^Department of Respiratory Medicine, Laurentius Hospital Roermond, Roermond, Netherlands

**Keywords:** lung cancer, lung neoplasms, brain metastasis, brain neoplasms, brain abscess, aspergillosis, differential diagnosis

## Abstract

In a patient with a medical history of cancer, the most probable diagnosis of an ^18^FDG-avid pulmonary mass combined with intracranial abnormalities on brain imaging is metastasized cancer. However, sometimes a differential diagnosis with an infectious cause such as aspergillosis can be very challenging as both cancer and infection are sometimes difficult to distinguish. Pulmonary aspergillosis can present as an infectious pseudotumour with clinical and imaging characteristics mimicking lung cancer. Even in the presence of cerebral lesions, radiological appearance of abscesses can look like brain metastasis. These similarities can cause significant diagnostic difficulties with a subsequent therapeutic delay and a potential adverse outcome. Awareness of this infectious disease that can mimic lung cancer, even in an immunocompetent patient, is important. We report a case of a 65-year-old woman with pulmonary aspergillosis disseminated to the brain mimicking metastatic lung cancer.

## Case Description

**Figure 1:**
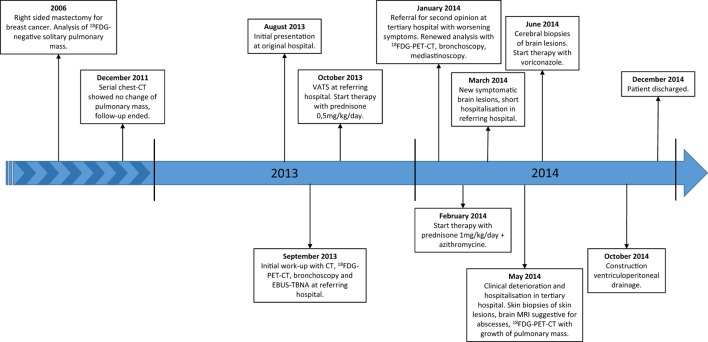
Timeline. Abbreviations: ^18^FDG, 18-fluordeoxyglucose; CT, computed tomography; VATS, video assisted thoracic surgery; ^18^FDG-PET-CT, 18-fluordeoxyglucose positron emission tomography-computed tomography; EBUS-TBNA, endobronchial ultrasound with transbronchial needle aspiration; MRI, magnetic resonance imaging.

A 65-year-old woman, never-smoker, was referred for a second opinion in January 2014 because of an abnormal computed tomography (CT) of the chest with a mass in the right lower lobe. Extensive evaluation in the referring hospital had not revealed a diagnosis. A clear overview of the medical disease history is demonstrated in a timeline (Figure 1). Her medical history consisted of a right-sided mastectomy for breast cancer in 2006, with no adjuvant treatment indicated. On the staging 18-fluordeoxyglucose positron emission tomography-computed tomography (^18^FDG-PET-CT) for the breast cancer in 2006 an asymptomatic, 30-mm diameter, lobulated ^18^FDG-negative solitary mass was seen in the right lower lobe. Bronchoscopic sampling for cytology and microbiological cultures showed neither proof of malignancy nor infection, and follow-up was chosen. Serial follow-up chest CTs up to December 2011 (total follow-up of 5 years) showed no change and follow-up was ended.

**Figure 2:**
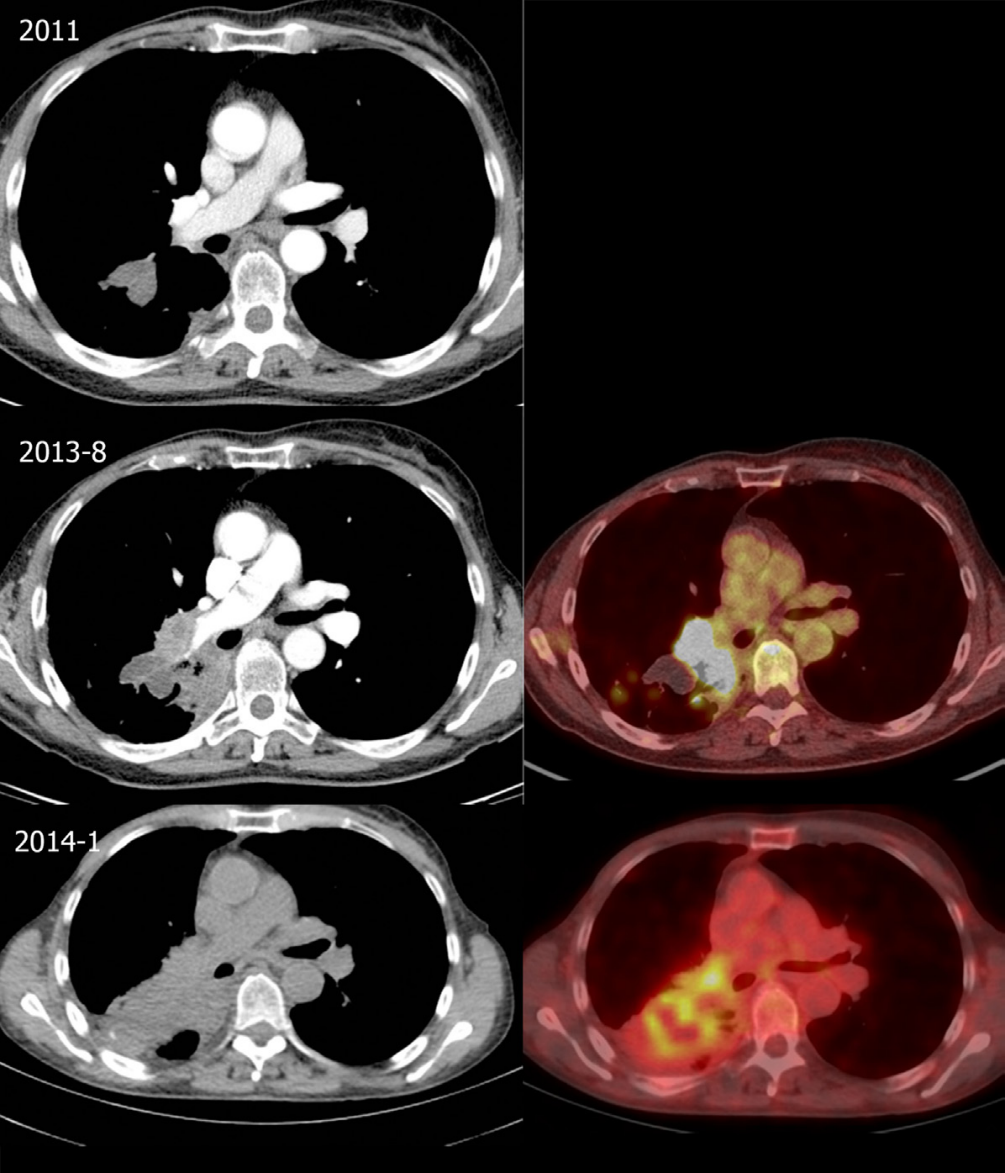
Evolution of thoracic lesions. Top: Follow-up chest computed tomography (CT) in 2011 showing a right-sided lobulated pulmonary mass at the right lower lobe (3.0-cm diameter). Middle: CT (left) and fusion 18-fluordeoxyglucose positron emission tomography-computed tomography (^18^FDG-PET-CT) (right) in August 2013 shows an increase at the medial side of the mass and right hilar lymphadenopathy, with intense 18-fluordeoxyglucose (^18^FDG) uptake. Bottom: CT (left) and fusion ^18^FDG-PET-CT (right) in January 2014 showing further growth of the ^18^FDG-avid mass in the right lower lobe with hilar invasion and a mild ^18^FDG-avid subcarinal lymph node.

**Figure 3:**
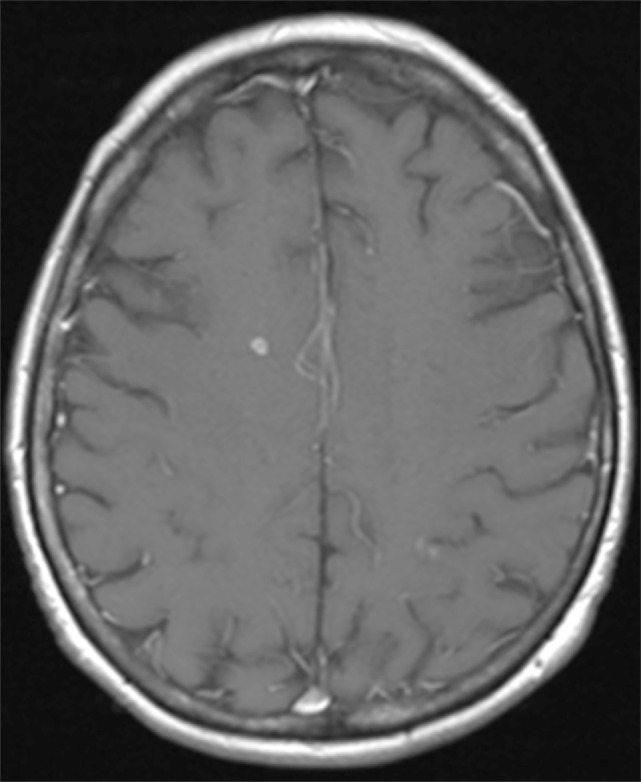
Brain magnestic resonance imaging in March 2014. T1-weighted image after gadolinium of the brain shows a small right frontal enhancing cerebral lesion.

**Figure 4:**
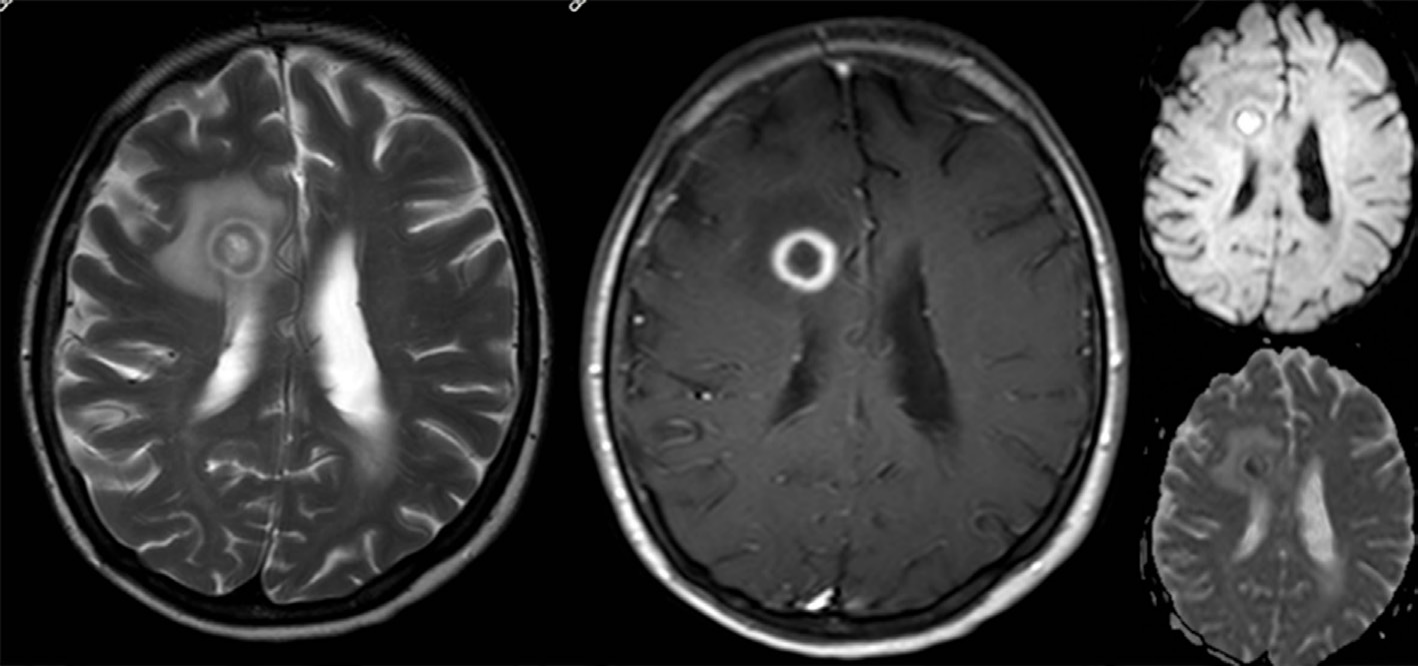
Brain magnetic resonance imaging in May 2014. There is an increase in size of the right frontal lesion with surrounding perilesional edema. T2-weighted image (left) demonstrates a hypo-intense rim with ring-enhancement after gadolinium (contrast-enhanced T1-weighted middle). At diffusion imaging (right panels) there is restricted diffusion in a part of the central area.

**Figure 5:**
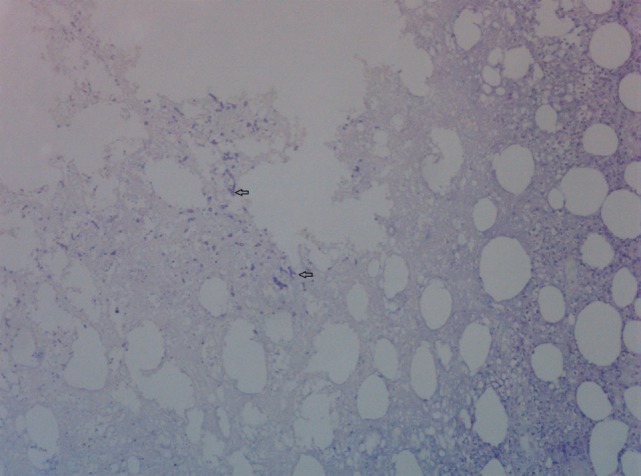
Skin biopsy with presence of fungal hyphae. Periodic Acid Schiff stain on skin biopsy with fungal hyphae stained purple. Two fungal hyphae with dichotomous branching (diagnostic of *Aspergillus*) are depicted (arrows).

**Figure 6:**
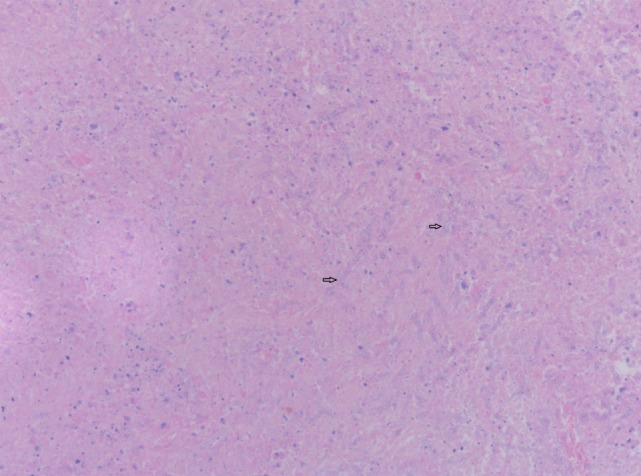
Cerebral biopsy with presence of fungal hyphae. Hematoxylin and eosin stain on cerebral biopsy showing nectrotic tissue with moderate numbers of septate fungal hyphae with parallel walls. Two fungal hyphae with dichotomous branching (diagnostic of *Aspergillus*) are depicted (arrows).

In August 2013, she was seen in the referring hospital because of a productive cough, dyspnea on exertion, tiredness, and weight loss since the last 3 months. There was no fever nor night sweats. ^18^FDG-PET-CT revealed an intense ^18^FDG-avid mass in the right lower lobe, just next to the old pulmonary lesion, with intense hilar lymphadenopathy (Figure 2). Differential diagnosis consisted of malignancy (metastatic breast cancer or primary lung cancer) or infection. Bronchoscopic sampling, CT-guided biopsy, and endobronchial ultrasound with transbronchial needle aspiration (EBUS-TBNA) revealed no malignancy and cultures were negative (Table 1). Transthoracic biopsy of the mass showed fibrosis and a chronic inflammation with histiocytic reaction. A video-assisted thoracoscopy with partial wedge resection of the pulmonary nodule was performed to obtain a definitive diagnosis and to rule out or confirm malignancy. Pathologic analysis of the resection specimen showed fibrosis with bronchiectasis, focal inflammation, and bronchiolisation of the alveoli, but no malignancy or microorganisms (Table 1). With a diagnosis suggestive of cryptogenic organizing pneumonia, prednisone 0.5 mg/kg/day was initiated, although, due to steroid side-effects, limited in dose and duration (prednisone 0.5 mg/kg/day for 3 weeks followed by gradual tapering of the dose, total duration of prednisone treatment was 2 months). Despite the steroids, her complaints worsened and the patient was referred to our tertiary center in January 2014. A new ^18^FDG-PET-CT showed progression of the ^18^FDG-avid lesion with extension into the mediastinum and lymph node station 4R, with also mass effect on the right pulmonary artery and invasion of the left superior pulmonary vein (Figure 2). There was no evidence of extrathoracic lesions. Because of the invasive growth, malignancy was again in the differential diagnosis. In our hospital, renewed analysis was performed with bronchoscopy and mediastinoscopy, both without evidence of malignancy or infection (Table 1). Cytology of the bronchial aspirate showed sporadic hyphae (most probable *Aspergillus*), but without growth on culture and these were considered contamination or colonization. In March 2014, a multidisciplinary decision was made for a treatment with a higher dose of prednisone 1 mg/kg/day in combination with macrolide antibiotic treatment for 3 months (with slow tapering of the steroids) under suspicion of cryptogenic organizing pneumonia, but without clinical or radiologic response. Additional diagnostics were considered for the growing part of the lesion, but a CT-guided biopsy and surgical sampling were both not possible because of the risk of a massive bleeding. A follow-up ^18^FDG-PET-CT was scheduled to evaluate whether in the follow-up lesions would become better accessible for further diagnostic work-up. During the pulmonary work-up, in March 2014, the patient developed new complaints of progressive muscle weakness and sensibility loss of the right upper arm. She was hospitalized in the referring hospital. Additional brain imaging with magnetic resonance imaging (MRI) revealed multiple brain lesions in the cortex and watershed region, in the left corpus callosum, the left thalamus and partially in the right semioval center, which were considered brain metastases by the referring hospital (Figure 3). The MRI was revised by an experienced neuro-radiologist in our hospital who withheld a differential diagnosis of ischemia and metastasis. The brain lesions were not accessible for a biopsy because of the location and small size. Because of a poor clinical condition combined with a differential diagnosis of ischemia, no brain radiation was initiated. Patient was discharged after 1½-week hospitalization in the referring hospital. The neuro-oncology multidisciplinary team advised follow-up MRI 3 months later. On this MRI (May 2014), two lesions had enlarged significantly with marked perilesional edema but other lesions showed shrinkage (Figure 4). The radiologic appearance with restrictive diffusion of these lesions on diffusion-weighted images was suggestive for (atypical) cerebral abscesses rather than metastases. The ^18^FDG-PET-CT showed further growth of the mass in the right lower lobe but without distant lesions. At the same time, the patient developed multiple ill-defined skin lesions. Because of her worse clinical condition, she was hospitalized in our tertiary hospital and cerebral and skin biopsies were performed: both showed marked inflammation and fungal hyphae with dichotomous branching, suggestive for *Aspergillus*, there was no evidence for malignancy (Table 1; Figures 5 and 6). Cultures of the wound fluid after skin biopsy also revealed *Aspergillus* (Table 1). The definitive diagnosis of proven invasive aspergillosis with pulmonary, mediastinal, cerebral, and skin involvement was made. Treatment with voriconazole was initiated with monitoring of serum and cerebral spinal fluid (CSF) voriconazole levels. Because of progressive somnolence caused by hydrocephalus, repeated CSF drainage was necessary. Eventually, five neurosurgical procedures were needed for effective control of the infection and adequate drainage of the CSF, with in the end placement of an internal ventriculo-peritoneal drain. During treatment with voriconazole, there was a slow clinical recovery. Additional immunological analysis did not reveal an immunity disorder; there were normal titers of total IgG, IgM, IgG, and IgA, there were no complement abnormalities, screening for antinuclear antibodies and antineutrophil cytoplasmic antibodies was negative. Only the use of prednisone could be identified as immunosuppressant factor which aided the further dissemination of this opportunistic infection. MRI of the brain performed after 6 months of treatment showed marked improvement without evidence of hydrocephalus, the chest CT also improved. Patient was discharged in December 2014, 7 months after admission. She rehabilitated and made a near complete recovery.

**Table 1:** Diagnostic test results.

Date	Specimen	Microbiological test results	Pathological test results
2006	Bronchial washing right lower lobe	Culture negative for bacteria and fungi Auramine-rhodamine stain negative	No arguments for malignancy

August 15, 2013	Bronchial (brushing and) washing right lower lobe	Culture negative for bacteria and fungi Auramine-rhodamine stain negative	Active inflammation No arguments for malignancy

September 03, 2013	Computed tomography-guided biopsy right lower lobe	NA	Fibrosis with anthracosis and chronic inflammation No arguments for malignancy

September 23, 2013	EBUS 10R	NA	Representative specimen of reactive lymph node without arguments for malignancy

October 29, 2013	Wedge resection apical segment right lower lobe	Culture negative for bacteria	Fibrotic node with extensive chronic inflammation and bronchialization of the alveoli No arguments for malignancy No arguments for actinomyces infection

October 29, 2013	Urine	Culture negative for bacteria and fungi	NA

November 04, 2013	Blood	Culture negative for bacteria and fungi	NA

November 07, 2013	Urine	Culture negative for bacteria and fungi	NA

November 07, 2013	Wound fluid chest drain entrance	Sporadic *S. aureus*	NA

January 20, 2014	Bronchial washing right lower lobe	Bacterial culture with commensal throat flora Culture negative for fungi Culture negative for actinomyces Culture negative for nocardia Auramine-rhodamine stain negative Culture negative for mycobacteria	Active inflammation, sparse fungal hyphae and bacteria No arguments for malignancy

January 30, 2014	Mediastinoscopy 4L and 7	NA	Lymph node tissue without evidence of malignancy Extensive sinushistiocytosis at lymph node station 7

March 31, 2014	Cerebrospinal tap	NA	No arguments for malignancy or infection

May 02, 2014	Skin biopsy	Bacterial culture with coagulase-negative staphylococci Fungal culture with *Verticillium* species and *Aspergillus fumigatus*	Extensive active inflammation with a lobular panniculitis and localization of fungal hyphae No arguments for malignancy

May 02, 2014	Wound fluid skin biopsy	Fungal culture with *A. fumigatus*	NA

May 13, 2014	Serum	Galactomannan negative HIV 1 and HIV 2 antigen and immunoglobulin negative *Toxoplasma gondii* IgG positive, IgM negative *Treponema pallidum* immunoglobulin negative	NA

May 13, 2014	Blood (×2)	Culture negative for bacteria and fungi	NA

May 13, 2014	Urine	Culture negative for bacteria and fungi	NA

May 19, 2014	Serum	*Cryptococcus neoformans* antigen negative	NA

May 26, 2014	Serum	Interferon-gamma release assay negative	NA

June 02, 2014	Cerebral biopsy	*Aspergillus fumigatus*	Cerebral material with lytic cell remnants, active inflammation and presence of fungi (preference for *Aspergillus*)

*NA, not available*.

## Discussion

We report our experience of the diagnostic dilemma in this patient with disseminated aspergillosis mimicking metastatic cancer. The ^18^FDG-avid pulmonary lesions were highly suspicious for malignancy, especially in a patient with a history of breast cancer. Even in the presence of brain lesions, this suspicion remained high, as these brain lesions were first thought to be of metastatic origin. Eventually skin and cerebral biopsies and wound cultures did reveal the definitive diagnosis of disseminated invasive aspergillosis.

As a tertiary center, patients are frequently referred to our center with presumed lung cancer. In a retrospective analysis of a tertiary US hospital, the majority of such patients were proven to have a neoplastic process, only 1.3% had an infection (1). Pulmonary aspergillosis mimicking lung malignancy remains rare and only sporadic case reports are available in literature (1–7). In addition, symptoms (such as malaise, weight loss, cough, and hemoptysis) are non-specific and are overlapping those of a pulmonary neoplasm. Moreover, pulmonary aspergillosis can present as an infectious pseudotumour with radiological appearance and features similar and indistinguishable from lung cancer. When clinical and radiological features are suspected for malignancy, it is of utmost importance to strive for a definitive histopathological diagnosis. Many minimal invasive techniques such as bronchoscopy, CT-guided biopsy or EBUS-TBNA are available today to obtain this histopathological diagnosis. If not amenable or feasible or histopathological diagnosis cannot be obtained, a surgical approach might be necessary. The differential diagnosis can be very challenging and perseverance for diagnostic accuracy can be a hard and exhaustive exercise, which is demonstrated in our case. Despite several attempts with noninvasive and invasive procedures to obtain a histopathological diagnosis, there was no clear evidence for malignancy or infection. Maybe in retrospect, the hyphae in the bronchial aspirate could have raised the suspicion for invasive aspergillosis, although this is very rare in an immunocompetent host such as our patient. In retrospective studies with immunocompromised patients with a diagnosis of invasive aspergillosis, cytological examination of bronchial washings had a sensitivity of 64.0%, specificity of 99.1%, and positive predictive value of 88.9% (8), However, predictive values depend upon the prevalence of disease in the population tested and our patient did not have an impaired immunity. Moreover, various species of *Aspergillus* spp. can colonize the airways, especially in patients with a chronic pulmonary disease, without any pathogenic consequences (as was thought to be the case in our patient), but they are also capable of causing several and severe types of disease as has been described in patients with bronchiectasis (9). Pathological features in the surgical specimen suggestive for cryptogenic organizing pneumonia made this case more complex. Furthermore, the suspicion of malignancy remained high, with further growth of her thoracic disease and development of brain lesions, suspicious for brain metastasis. Indeed, cerebral abscesses caused by *Aspergillus* spp. can also mimic cerebral metastasis. Contrast-enhanced CT and MRI are the modalities of choice for imaging of the brain when brain metastasis is suspected, with MRI more appropriate in characterizing lesions (10–13). Although many MR features have been described, the differentiation between abscesses and necrotic brain tumors cannot be made in many cases with conventional MR imaging since its signal appearance can be similar to that of a cystic or necrotic tumor on conventional series (14, 15). A combination with diffusion-weighted MRI has been shown to be useful in the diagnosis of acute cerebral ischemia, malignancy, abscesses, cysts, and various forms of white matter disorders (16). In our case, the dissociated response, the hypo-intense rim at T2-weighted imaging and the diffusion-weighted MRI aided toward an infectious diagnosis. Previous reports in literature of a cerebral *Aspergillus* abscess mimicking a solid tumor are sparse; we could only identify two case resports (15, 17).

Coexistence of infectious pseudotumours and solid tumors at initial diagnosis have previously been reported (18), but are rather rare, especially in an immunocompetent patient. However, an endobronchial aspergilloma is thought to be able to infect endobronchial cancer lesions (19, 20). In general, most cases of coexisting infectious pseudotumours and lung cancer are rather a consequence of treatments with corticosteroids and/or chemotherapy.

## Conclusion

Pulmonary aspergillosis, even in the presence of cerebral abscesses, can present as an infectious pseudotumour with clinical and imaging characteristics resembling lung malignancy. These clinical and radiological similarities can cause significant diagnostic difficulties, with a subsequent therapeutic delay and a potential adverse outcome. A definitive histopathological diagnosis should always be strived for when malignancy is suspected, but awareness that this infectious disease can mimic lung cancer even in immunocompetent patients is of great diagnostic and prognostic importance.

## Ethics Statement

Patient gave written informed consent in accordance with the Declaration of Helsinki.

## Author Contributions

Conception and design and drafting of the manuscript: MV, AD, and LH. Drafting the manuscript for important intellectual content: all authors. All co-authors critically revised the article and gave final approval of this version to be published.

## Conflict of Interest Statement

MV has nothing to disclose. AD: none related to current manuscript. Outside of the submitted work: advisory boards: Roche, Pfizer, Eli Lilly Boehringer Ingelheim, MSD, Astra Zeneca, BMS; fees for lectures: BMS, Roche, Ely Lilly. VS: none related to current manuscript. KV: none related to current manuscript. AP: none related to current manuscript. Outside of the submitted work: fees for lectures: Bayer. AL: none related to current manuscript. CP has nothing to disclose. LH: none related to current manuscript. Outside of the submitted work: advisory boards: Boehringer Ingelheim and BMS; fees for lectures: MSD, Astra Zeneca, Roche, research grant: Roche.
